# Proteasomal Degradation of p53 by Human Papillomavirus E6 Oncoprotein Relies on the Structural Integrity of p53 Core Domain

**DOI:** 10.1371/journal.pone.0025981

**Published:** 2011-10-27

**Authors:** Xavier Bernard, Philip Robinson, Yves Nominé, Murielle Masson, Sebastian Charbonnier, Juan Ramon Ramirez-Ramos, Francois Deryckere, Gilles Travé, Georges Orfanoudakis

**Affiliations:** 1 Oncoprotéines, UMR 7242 CNRS, Ecole Supérieure de Biotechnologie de Strasbourg, Université de Strasbourg, Illkirch, France; 2 Pharmaco-Epidémiologie, CHU de Bordeaux, INSERM CIC 0005, Université de Bordeaux, Bordeaux, France; University of Michigan, United States of America

## Abstract

The E6 oncoprotein produced by high-risk mucosal HPV stimulates ubiquitinylation and proteasome-dependent degradation of the tumour suppressor p53 via formation of a trimeric complex comprising E6, p53, and E6-AP. p53 is also degraded by its main cellular regulator MDM2. The main binding site of p53 to MDM2 is situated in the natively unfolded N-terminal region of p53. By contrast, the regions of p53 implicated in the degradation by viral E6 are not fully identified to date. Here we generated a series of mutations (Y103G, Y107G, T155A, T155V, T155D, L264A, L265A) targeting the central folded core domain of p53 within a region opposite to its DNA-binding site. We analysed by *in vitro* and *in vivo* assays the impact of these mutations on p53 degradation mediated by viral E6 oncoprotein. Whereas all mutants remained susceptible to MDM2-mediated degradation, several of them (Y103G, Y107G, T155D, L265A) became resistant to E6-mediated degradation, confirming previous works that pointed to the core domain as an essential region for the degradation of p53. In parallel, we systematically checked the impact of the mutations on the transactivation activity of p53 as well as on the conformation of p53, analysed by Nuclear Magnetic Resonance (NMR), circular dichroism (CD), and antibody probing. These measurements suggested that the conformational integrity of the core domain is an essential parameter for the degradation of p53 by E6, while it is not essential for the degradation of p53 by MDM2. Thus, the intracellular stability of a protein may or may not rely on its biophysical stability depending on the degradation pathway taken into consideration.

## Introduction

The p53 protein is a key regulator of cell proliferation. In non-dividing cells, p53 is tightly maintained at a steady-state level, which decreases when cells enter S-phase to undergo proliferation [1], [2]. Various factors of deregulated cell proliferation, such as genotoxic stress or oncogene expression, induce both the accumulation of p53 and the modulation of its activities [3]. This results in activation of a variety of molecular pathways leading either to cell-cycle arrest, senescence, or apoptosis. Due to its central role in cell cycle regulation, p53 is systematically deregulated in cancers and other pathologies involving abnormal cell proliferation.

**Figure 1:**
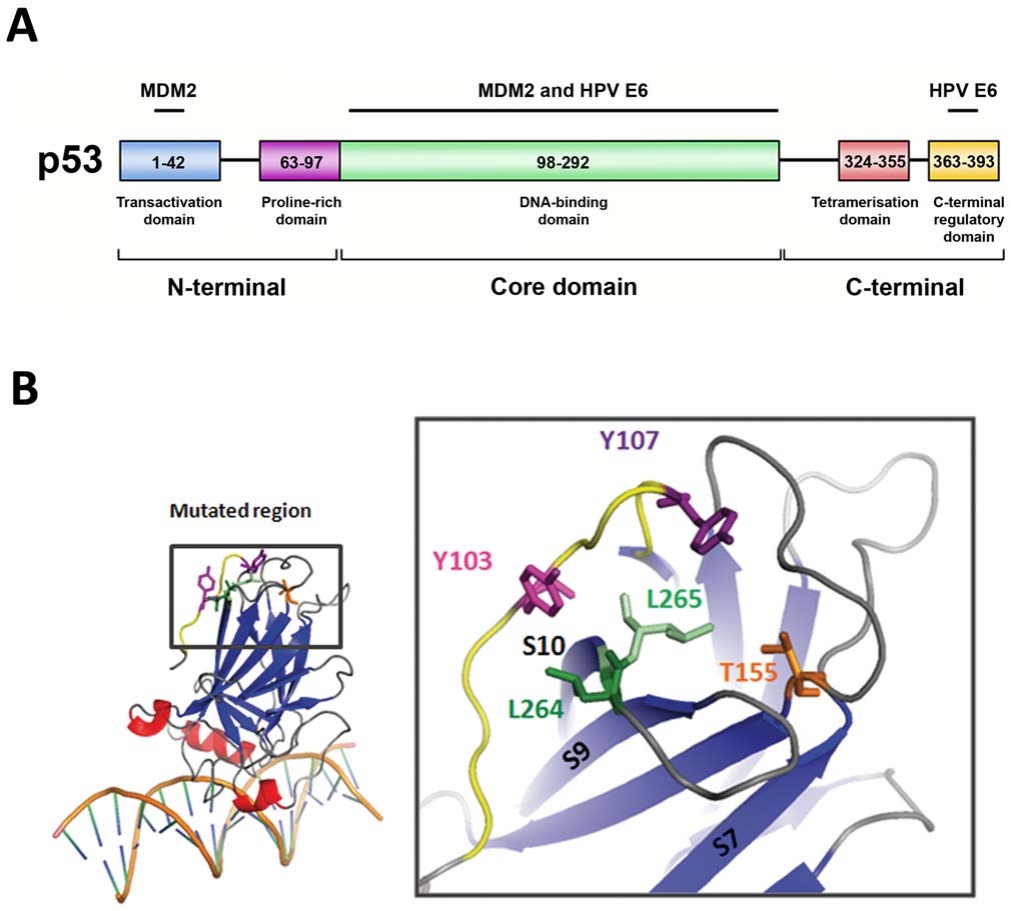
Localization of residues within the structure of p53 core domain. (A) Schematic view of the domain structure of p53. The 393-residue p53 protein comprises an N-terminal transactivation domain (blue), followed by a proline-rich region (purple), a central DNA-binding core domain (green), a tetramerization domain (red) and a regulatory domain (yellow) at the extreme C-terminus. The regions of possible interaction between p53 and MDM2 or p53 and HPV E6 are indicated. (B) Enlarged view of the three-dimensional structure of p53 core domain. Mutants analysed for this study are all localised in the same tridimensional region, distal from the DNA binding site. The leucine 265 is shown in light green, the leucine 264 in dark green, the threonine 155 in orange, the tyrosine 103 in pink, the tyrosine 107 in purple and the region in yellow corresponds to the residues 99 to 107. The β-strands are shown in blue (S7, S9 and S10) and the a-helix in red. The view was created from PDB entry: 1TSR using the PyMOL software.

P53 is a multifunctional 393-residue protein (fig. 1A). The central core domain (residues 98–292) constitutes a single folded unit that bears the sequence-specific DNA-binding activity of p53. The core domain is flanked by modulatory regions including an N-terminal transactivation domain (residues 1–42), a proline-rich domain (63–97), a helical tetramerisation domain (324–355) and a C-terminal regulatory domain (363–393). These regions modulate the transcriptional activity of the core domain in response to various ligands (proteins, damaged DNA) or modifications such as phosphorylation or acetylation [4]. The three-dimensional structure of the core domain bound to its double-stranded DNA target has unravelled the structural basis of p53 transcriptional activity [5].

Genotoxic stress-induced p53 accumulation mainly results from post-translational stabilisation [6]–[8]. Therefore, both the physiological and pathological mechanisms of p53 turnover have been extensively studied. The cellular MDM2 protein, first known as a transcriptional target of p53, was found to act as a E3 ubiquitin-ligase, which transfers ubiquitin (Ub) onto p53, thereby targeting it to proteasome-mediated degradation [9], [10]. Perturbation of MDM2 function leading to enhanced degradation of p53 is a key event in numerous cancers [11]–[14]. Enhancement of p53 degradation has also been recognised as one of the strategies used by oncoviruses that stimulate cell proliferation for the sake of their own life cycle [15]. Adenoviral proteins E1b 55k and E4 34k have been shown to form a stable complex with p53 leading to its enhanced ubiquitination and subsequent proteasome-mediated degradation [16], [17]. The E6 oncoprotein of “high-risk” HPV types has also been demonstrated to catalyse the ubiquitin-mediated degradation of p53 [18]–[20]. E6 is a small protein of about 150 residues composed of two 70-residue zinc-binding domains [21]–[23]. E6 interacts with E6AP, a 850-residue E3 ubiquitin-ligase [24]–[26]. E6AP contains an E6-binding site within a central 18-residue stretch comprising the “LxxLL” motif that is found in several other targets of E6 [27], [28]. p53 has been proposed to contain two distinct E6-binding sites [29]–[31]. The C-terminal modulatory domain (residues 356–393) has been suggested to contain a primary E6-binding site recognised by all E6 proteins of both low- and high-risk HPV types, but this interaction has no role in E6-induced p53 degradation [32], [33]. The core domain of p53 appears to contain a secondary E6-binding site restricted to E6s of high-risk HPVs. E6 binding to this secondary site is E6AP-dependent and is required for p53 ubiquitination and subsequent degradation [34]. The precise location of this site within the p53 core domain structure is still unknown.

Li and Coffino [30] showed that aminoacids 100–150 of p53 contain a degradation domain and hypothesized this region as the second binding site for high risk E6. Additionally, Gu *et al.* [29] proposed that p53 has a unique sequence element within the core domain (aminoacids 92–112) that acts as a signal for MDM2-mediated degradation and the binding of oncoproteins, which direct p53 degradation towards proteasome pathway. This secondary site was also required for E6/E6AP-mediated p53 ubiquitination and subsequent degradation, whereas the primary site was dispensable for these activities [29]. More recently a secondary MDM2 binding site was suggested to exist in the flexible loop which links S9–S10 β-strands of the p53 core domain and proposed as a regulatory element modulating p53 ubiquitination [35]. NMR and fluorescence anisotropy measurements have also suggested that a second MDM2 binding site is located within p53 core domain [36]. On the other hand, Bech-Otschir *et al.* [37] observed that phoshorylation of T155, a threonine residue also situated in the core domain, by the COP9 signalosome (CSN) appeared to be important for p53 degradation by the Ub-26S proteasome system and that replacement of T155 by valine stabilized p53 in HeLa cells.

Remarkably, when the above-mentioned mutations are plotted on the structural model of p53, it can be observed that they are all focused on the same region of the p53 core domain. A fragment of the region defined by Gu *et al.* in 2001 [29], spanning approximately residues 99–107, forms an extended N-terminal tail structure that runs on the surface of the core domain. This N-terminal tail is anchored to the rest of the core structure via aromatic residues Y103 and Y107, which interact with residues L264 and L265 that belong to the loop connecting β-strands S9 and S10 (fig. 1B). Moreover, the amino acid T155 highlighted by Bech-Otschir *et al.* in 2001 [37] is also located in the vicinity of these residues.

In the present work, we generated p53 constructs bearing various mutations on key residues situated within the above mentioned region of p53 core domain. E6 and MDM2 dependent degradation and transactivation activity of the mutated p53 proteins were analysed both *in vitro* within cell extracts and *in vivo* within a p53-null cell line. In addition, the incidence of the mutations on the structural integrity of p53 was studied by circular dichroism (CD) and NMR ^1^H-^15^N correlation spectroscopies. While mutants T155A, T155V and L264A were still degraded by E6, the mutants Y103G, Y107G, T155D and L265A became resistant to E6 mediated degradation. By contrast, all mutants were still degraded by MDM2. Among the E6-resistant mutants, Y107G, T155D and L265A appeared to have completely lost the native p53 fold, whereas Y103G displayed a folded yet significantly altered conformation.

## Results

### Mutation L265A protects p53 from E6-mediated degradation

**Figure 2:**
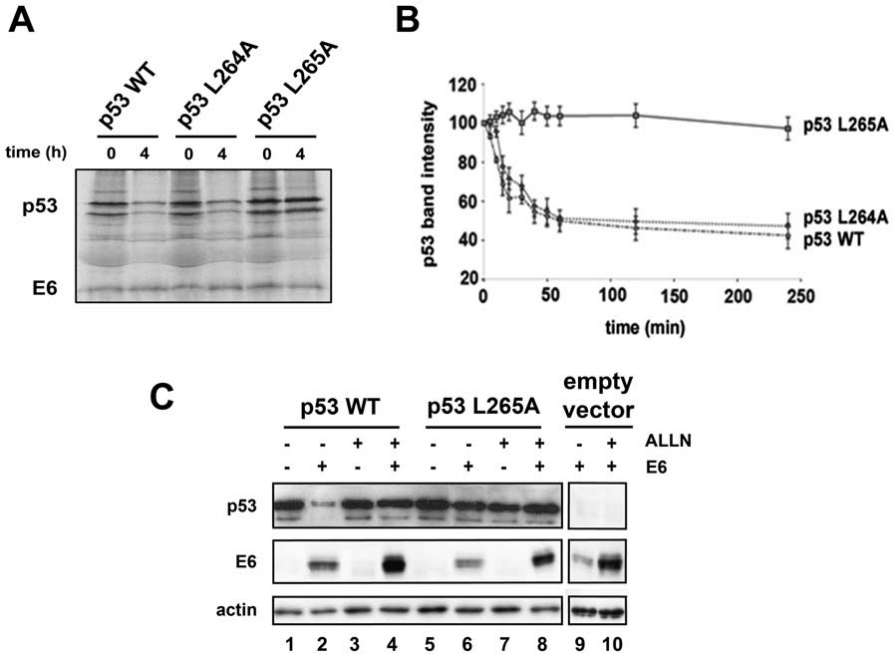
Degradation of p53 wild type and indicated mutants via E6. (A) Radiolabeled p53 (wild type and indicated mutants) and HPV16 E6 proteins produced in rabbit reticulocyte lysates were incubated together at 28°C. Aliquots were removed at the indicated times before separation by 12% SDS-PAGE and exposition to photographic film. (B) Same experiment as in A, but levels of radiolabeled p53, after exposition to photographic film, were quantified by densitometry (BIO-RAD, Quantity One Software). Mean values (AU) ± standard deviation for three independent experiments are shown. (C) H1299 cells were co-transfected by vectors for transient expression of HPV16 E6 and p53 proteins. Where indicated, 4 h prior to harvesting, the medium was supplemented with the 26S proteasome inhibitor ALLN at a final concentration of 100 µM. 24 h after transfection, extracted proteins were separated by 12% SDS-PAGE and analysed by Western-blotting using monoclonal anti-p53 antibody, polyclonal anti-actin antibody and monoclonal anti-16-E6 antibody.

According to observations made by Shimizu *et al.* [35], the aminoacids S261, L264 and F270 of p53 seem to be implicated in the MDM2-dependent ubiquitination. On the other hand, residues L264 and L265 are spatially close to the sequence segment 92–112 that has been previously described as an important region for the E6 mediated degradation via the 26S proteasome [29]. This prompted us to generate two single-point mutants of p53: p53-L264A and p53-L265A. HPV16 E6 as well as the wild-type (WT) and mutant p53 proteins were synthesised as ^35^S-labeled proteins in a rabbit reticulocyte lysate translation system. Lysates containing the appropriate labeled proteins were then combined to analyse the E6-mediated degradation of the p53 mutants *in vitro*. p53-WT along with p53-L264A were efficiently degraded in the presence of HPV16 E6, whereas p53-L265A was found to be resistant to degradation in the same conditions (Fig. 2A and B). Mutant L265A was also found to be resistant to HPV16 E6 degradation *in vivo*, in H1299 p53-null cells co-transfected with vectors coding for the p53 mutant and HPV16 E6 protein (Fig. 2C). Remarkably, in this assay the E6 cellular levels were strongly increased in the presence of the proteasome inhibitor ALLN (Calpain inhibitor I) (Fig. 2C, compare intensity of E6 band between lanes 3 and 4 or between lanes 7 and 8), and this effect was also observed in the absence of p53 (Fig. 2C, compare intensity of E6 band between lanes 9 and 10). This result is consistent with previous observations [38] demonstrating that E6 is ubiquitinated and degraded by the proteasome.

### Mutations Y103A and Y107G protect p53 from E6 degradation

**Figure 3:**
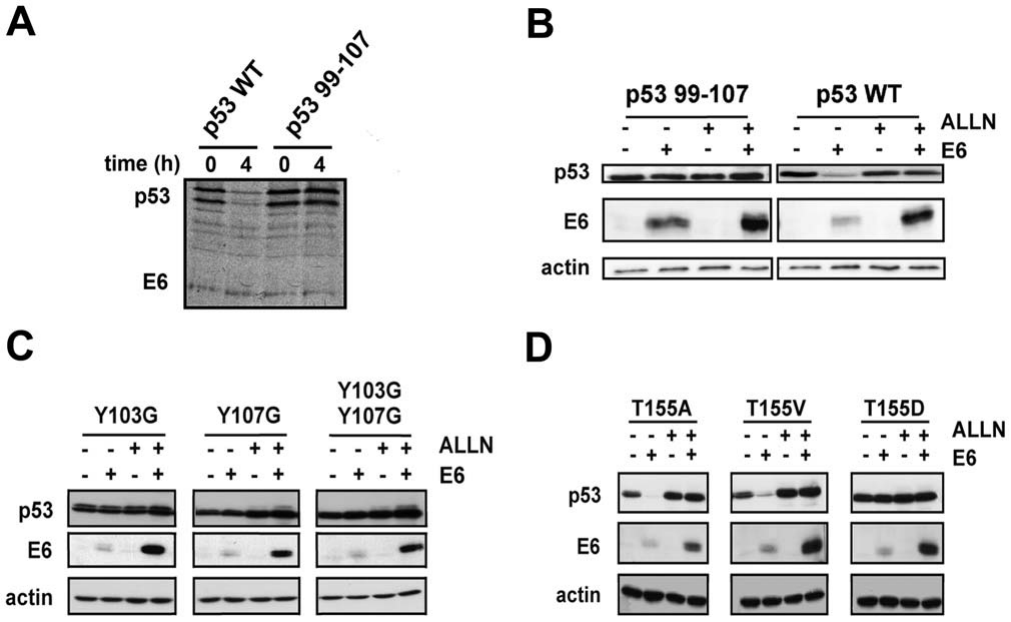
Behaviour of p53 mutants in presence of E6. (A) Radiolabeled p53 (wild type and indicated mutants) and HPV16 E6 proteins produced in rabbit reticulocyte lysates were incubated together at 28°C. Aliquots were removed at the indicated times before separation by 12% SDS-PAGE and exposition to photographic film. (B, C and D) H1299 cells were co-transfected by vectors for transient expression of HPV16 E6 and p53 proteins. Where indicated, 4 h prior to harvesting, the medium was supplemented with the 26S proteasome inhibitor ALLN at a final concentration of 100 µM. 24 h after transfection, extracted proteins were separated by 12% SDS-PAGE and analysed by Western-blotting using monoclonal anti-p53 antibody, polyclonal anti-actin antibody and monoclonal anti-16-E6 antibody.

The sequence segment 92–112 that has been previously described as an important region for the E6 mediated degradation via the 26S proteasome [29]. Furthermore, residues Y103 and Y107 of this segment are spatially close to residue L265 which we have just identified as a key residue for E6 mediated p53 degradation (Fig. 1B). We first generated the multiple mutant p53-99-107 in which the sequence ^99^ SQKTYQGSY ^107^ was replaced by the sequence ^99^ GAGAGAGAG ^107^. Similar to p53-L265A, this mutant was resistant to HPV16 E6-mediated degradation both *in vitro* and *in vivo* (Fig. 3A and 3B). We then generated single point mutants in which Y103 and Y107 were substituted by glycine. Both p53-Y103G and p53-Y107G were resistant to HPV16 E6-mediated degradation *in vivo* (Fig. 3C).

### Mutation T155D but not T155A or T155V, protects p53 from degradation by HPV16 E6

The phoshorylation of T155 can modulate p53 stability via the COP9 signalosome (CSN), which participates in p53 ubiquitination. The phoshorylation state of T155 was also described as important in high-risk E6 induced degradation of p53 [37]. We generated mutants p53-T155V, p53-T155A and p53-T155D. The replacement of T155 by alanine or valine aimed to inactivate the putative phosphorylation site, whereas its replacement by aspartate aimed to mimic a constitutively phosphorylated threonine. The mutant proteins p53-T155A and p53-T155V were degraded in presence of HPV16 E6 in transfected H1299 cells, whereas p53-T155D was resistant to degradation mediated by E6 (Fig. 3D).

### All p53 core mutants are still degraded by MDM2

**Figure 4:**
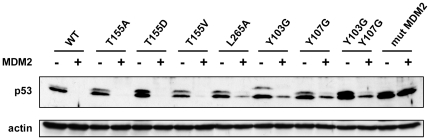
MDM2-mediated degradation of p53 mutants. H1299 cells were co-transfected by vectors for transient expression of MDM2 and p53 proteins. 24 h after transfection, extracted proteins were separated by 10% SDS-PAGE and analysed by Western blotting using monoclonal anti-p53 antibody, polyclonal anti-actin antibody.

We also examined whether the various mutations altered the MDM2-dependent degradation of p53 in H1299 cells. The major binding site of MDM2 on p53 is located in the N-terminal transactivation domain (residues 14–27) [39], [40]. A secondary putative MDM2-binding site has been reported in the p53 core domain which seems to play a regulatory role in modulating p53 ubiquitination [35], [36], [41]. A vector expressing MDM2 was cotransfected with vectors expressing the different mutants of core domain of p53 full length in H1299 cells. All mutated proteins were degraded in the presence of MDM2 demonstrating that none of these mutations prevented MDM2 to bind and to degrade p53. However, L265A, Y103G, Y107G, and the double mutant Y103G/Y107G appeared slightly less sensitive to MDM2-mediated degradation as compared to p53-WT (Fig. 4). As a control, we used a vector encoding mutant p53-mutMDM2, bearing the multiple mutations F19A/W23A/L26A altering the primary binding site of p53 to MDM2. In contrast to all the core domain mutants, p53-mutMDM2 was fully resistant to MDM2-mediated degradation (Fig. 4).

### Mutations Y107G, T155D and L265A, but not T155A, T155V and Y103G, fully disrupt the capacity of p53 to transactivate the p21 promoter

**Figure 5:**
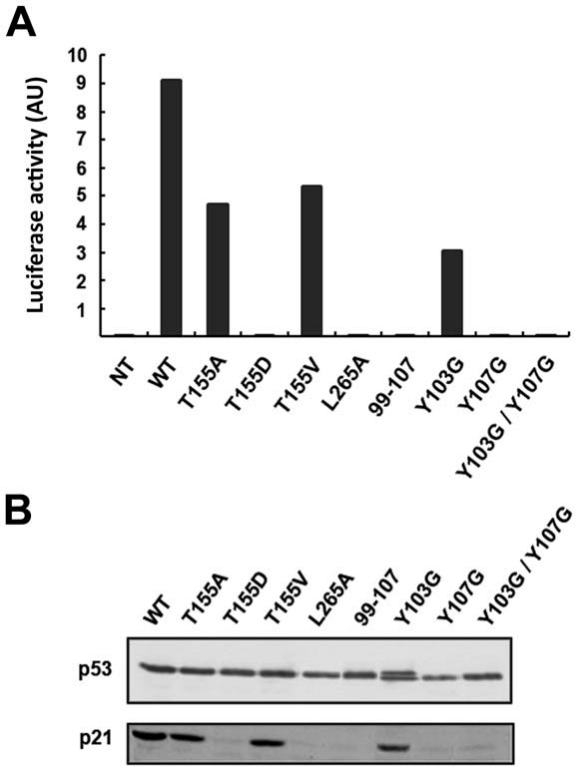
Transactivation activity of p53 mutants. (A) H1299 cells were co-transfected by vectors coding for p53 proteins and a vector expressing the reporter gene luciferase under the control of the p21 promoter (NT: Not Transfected). Crude cellular extracts were then analysed by luminescence dosage (AU: Arbitrary Units). (B) H1299 cells were co-transfected by vectors for transient expression p53-WT and mutant proteins. 24 h after transfection, crude extracts were separated on 12% SDS-PAGE and analysed by Western blotting using monoclonal anti-p53 antibody, anti-p21 antibody.

Next, we tested the ability of the p53 mutants to transactivate the p21 gene promoter. First, the transactivation capacities of WT and mutated p53 proteins were examined in the presence of the p21 gene promoter using a luciferase reporter construct. The p53-T155A, p53-T155V, and p53-Y103G mutants remained able to transactivate the p21 luciferase reporter construct while p53-Y107G, p53-T155D and p53-L265A failed (Fig. 5A). To confirm these results, p53 mutant proteins were expressed ectopically in H1299 cells and crude extracts were submitted to SDS-PAGE and Western blot analysis. In H1299 cells, the expression of endogenous p21 protein was induced in the presence of p53-WT, p53-Y103G, p53-T155A or p53-T155V, but not in presence of p53-Y107G, p53-T155D or p53-L265A (Fig. 5B).

L265 belongs to the loop connecting strands S9 and S10, which is distal from the DNA-binding surface [42]. T155 and Y107 are also distal from the DNA binding domain of p53. Therefore we can exclude that the mutagenesis of these residues altered the transactivation property by directly modifying the DNA-binding site. More likely, the mutations T155D, L265A, and Y107G may have introduced conformational changes in the core domain which indirectly disrupted the DNA binding activity of p53, subsequently inactivating the capacity of p53 to induce p21 gene transactivation.

### p53-Y107G, p53-T155D and p53-L265A exhibited a structural mutant conformational state

To investigate whether the Y107G, T155D and L265A mutations altered the fold of p53, we used anti-p53 antibodies known to distinguish between “native” (i.e., wild-type) and “mutant” (i.e., conformationally altered) conformations of p53 core domain [43].

**Figure 6:**
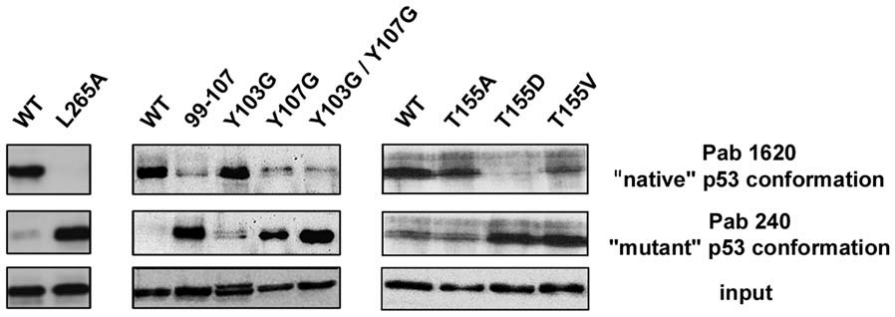
Conformation of p53 mutants. H1299 cells were transfected by vectors for transient expression of p53 proteins. Cell lysates were incubated with mouse monoclonal anti-p53 antibody, either the Pab 1620 recognising only the “wild-type” conformation epitope or the Pab 240 recognising only the “mutant” conformation epitope. Immune complexes and whole lysates (input) were separated by 12% SDS-PAGE and subjected to Western blotting using antibodies against p53 (polyclonal rabbit).

The Pab 1620 antibody recognises preferentially wild-type p53 with native fold whereas the Pab 240 antibody recognises a buried epitope on β-strand S7, which becomes accessible only upon unfolding of this region [43], [44]. Both p53-WT and p53 mutants were transiently expressed in H1299 cells and the crude extracts immunoprecipitated using either Pab 1620 or Pab 240 antibody and detected by Western blot analysis (Fig. 6). p53-WT was recognised exclusively by Pab 1620 antibody, indicating that it was in its native conformation state. Similarly, the p53-Y103G and the p53-T155A point mutants bound predominantly to Pab 1620 antibody which selectively recognises native p53. p53-T155V is recognised by both antibodies which suggests a mixture of native and non-native conformation. p53-Y103G is of particular interest as this mutant has lost the capacity to be degraded by E6, yet seems to retain a native-like conformation since it is preferentially recognised by Pab 1620 antibody. By contrast, p53-L265A, p53-Y107G and p53-T155D were predominantly recognised by Pab 240 suggesting that they have lost the native p53 conformation.

### The mutant p53-Y103G core domain displayed a native conformation

In order to confirm the observations obtained with conformation-sensitive antibodies, core domains of the human p53-WT protein (p53core-WT), and the mutants p53-L265A (p53core-L265A) and p53-Y103G (p53core-Y103G) were overexpressed in *E. Coli*, purified and analysed by biophysical methods. Then, the biophysical status of p53core-L265A and p53core-Y103G mutants was examined and compared to p53core-WT.

**Figure 7:**
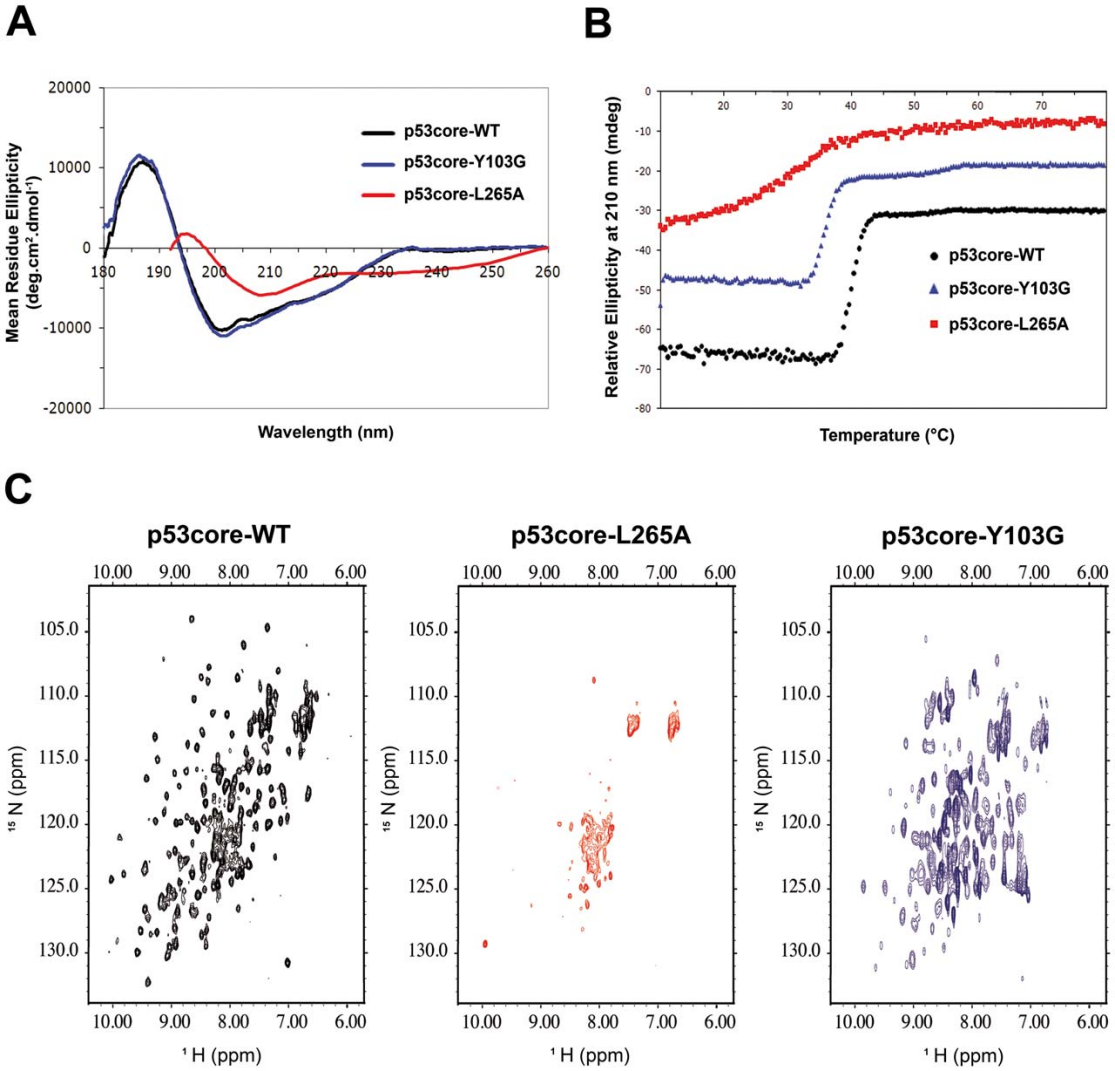
Biophysical analysis of Wt and mutant p53 core domain. (A) Comparison of different p53 core domain proteins (WT, L265A, Y103G) with respect to secondary structure content by CD. The spectra were recorded in 20 mM sodium phosphate (pH 6.8), 20 mM NaCl, 2 mM DTT at 10°C. The far-UV spectrum of the p53wt and p53Y103G are similar and show characteristics of folded proteins with a minimum at 201 nm while the spectrum of the L265A p53 core domain suggests a large proportion of unfolded protein as indicated by the shift of the minimum towards smaller wavelength and a negative signal at 200 nm. (B) Thermal denaturation of the p53 core domain proteins monitored by far-UV CD spectroscopy at 210 nm. The spectra were recorded in 20 mM sodium phosphate (pH 6.8), 50 mM NaCl, 2 mM DTT. For clarity, spectra have been offset by 10 mdeg between each curve. (C) ^1^H-^15^N correlation spectra of p53 core domain (residues 94–312) acquired at 10°C on a Bruker DRX600 spectrometer equipped with a z-gradient triple resonance cryoprobe. The p53WT core domain is represented in black (left panel), the p53L265A core domain in red (middle panel) and the p53Y103G core domain in blue (right panel).

We first recorded far-UV CD spectra of the three purified samples (Fig. 7A). Usually, far-UV CD spectra are recorded in order to estimate secondary structure contents. An accurate estimate of contents requires recording data at wavelength as low as possible and at least below 192 nm. However, we were not able to reach such a low wavelength with the p53core-L265A sample due to the relatively high salt concentration that was required for the stability of the sample. Nevertheless, comparison of CD spectra of mutated p53 core domain samples with WT is sufficient to know whether the secondary structure content is perturbed or not. P53core-WT and p53core-Y103G spectra could be well superimposed, suggesting a similar secondary structure content (Fig. 7A). By contrast, the p53core-L265A spectrum dramatically changed compared to WT, suggesting that mutation strongly altered the conformation of the core domain.

Thermal denaturation was also assessed in order to check and compare thermal stability of the domains (Fig. 7B). Thermal denaturation midpoints were 40 and 36°C for p53core-WT and p53core-Y103G domains respectively, suggesting that the p53core-Y103G mutant was thermodynamically destabilised as compared to wild-type p53. However the thermal transition for the p53-Y103G core domain mutant remained sharp suggesting that this mutant remains cooperatively folded.

By contrast, the denaturation curve of the p53core-L265A mutant displayed a very broad non cooperative transition centred around 25°C. This observation suggests that p53core-L265A is unfolded, displaying mainly random conformations.

To further analyse the effect of the mutations on p53 conformation, and validate the observations obtained with antibodies, we produced ^15^N-labeled samples of p53 core domain. The NMR ^1^H-^15^N correlation spectrum of the p53core-WT core domain recorded by NMR spectrometry matched formerly published spectra (for reference and peak assignment see Wong *et al.* [45]) (Fig. 7C). The p53core-WT spectrum displayed a well dispersed peak pattern with a homogeneous linewidth characteristic of a folded protein. By contrast, the spectrum of the p53core-L265A had lost the characteristic peak distribution of wild-type domain, and instead displayed signals in the region typical for random coil conformation, together with a decrease in signal intensities as well as linewidth broadening. These observations indicated that the domain was in a non-native conformation (for a reference showing a HSQC spectrum of unfolded p53core-WT see Rüdiger *et al.* [46]). However the purified p53-L265A core domain was soluble and monodisperse after gel filtration and could be concentrated to relatively high concentration for NMR investigation. p53core-T155V and p53core-Y107G point mutants also displayed spectral characteristics of non-folded proteins, although their purification and concentration resulted more difficult than for p53-L265A core domain (data not shown).

The ^1^H-^15^N correlation spectrum of p53-Y103G mutant core domain displayed a dispersed peak pattern characteristic of a folded protein, and retained at least 80% of the cross peaks observed for the p53-WT core domain. However, the linewidth of the cross peaks was more heterogeneous than for the wild-type protein, suggesting that some regions in the mutant may be prone to conformational exchange and/or self-association. In addition, close to 20% of the original cross-peaks of the WT core domain were not found, whereas some very intense additional peaks appeared in a region characteristic for residues in random conformations (8 to 8.5 ppm). Interestingly, most of the cross peaks of the p53-WT spectrum which were no more observed in the mutant p53core-Y103G spectrum corresponded to residues of the β-strand S10 directly in contact with the loop 92–112 described by Gu *et al.* [29] and containing the Y103 and Y107 residues. These observations suggest that the p53-Y103G mutant core domain is folded yet presents significant conformational alterations as compared to the wild type core domain. Most probably, these conformational alterations mainly concern residues spatially close to residue 103, i.e. residues situated in the N-terminal segment and in β-strand S10.

## Discussion

In the present work we studied the importance of residues within a region of p53 core domain that has been reported to contain a binding site for E6 from high-risk HPVs [29], [30], [37], and MDM2 [35], [36], [41]. Consistent with previous reports where chimeras of p53 with the 92–112 region from p73 were resistant to HPV16 E6-mediated degradation [29], we observed that multiple point mutagenesis of the strech 99–107 was sufficient to prevent p53 degradation in the presence of HPV16 E6 both *in vitro* and *in vivo*. In the structure of p53 core domain Y103 and Y107 are in contact with L264 and L265 situated in the linker between S9 and S10 segments. The single point mutation of Y107 to glycine protected p53 from HPV16 E6-mediated degradation, probably due to the alteration of the fold of the core domain. Indeed, mutant Y107G failed to transactivate the p21 promoter and it did not bind to the Pab 1620 antibody that selectively recognises the folded conformation of p53, whereas it bound to the Pab 240 antibody directed against a buried epitope on β-strand S7 (residues 212–217), which becomes accessible upon unfolding of this region. On the other hand, replacement of Y103 by glycine was found to protect from the E6-mediated degradation, and although it impacted on the conformation of the resulting protein, it did not disrupt its ability to fold.

The replacement of amino acid L264, reported to be implicated in the ubiquitination of p53 by MDM2 [35], to alanine had no effect on p53 degradation in the presence of HPV16 E6 or MDM2 (data not shown). However, replacement of L265 to alanine protected p53 from HPV16 E6-mediated degradation *in vitro*. Interestingly, when transiently expressed in H1299 p53-null cells, p53-L265A mutant retained its susceptibility to MDM2-mediated degradation but resisted to HPV16 E6-mediated degradation and displayed an impaired transcriptional activation capacity. Further investigation using Pab 240 and Pab 1620 [43] showed that p53-L265A had a “mutant” conformation. Both CD and NMR results showed that the p53core-L265A had a mainly unfolded conformation. Taken together these results show that a p53 mutant possessing an unfolded core domain remains capable of interacting with and be degraded by MDM2. However, we noticed that p53-L265A, together with p53-Y103G and p53-Y107G, were less efficiently degraded by MDM2 as compared to p53 wild-type. This may be explained by a decreased binding of MDM2 to its putative target site within the core domain [47], which may influence the ubiquitination level of p53 [35]. Alternatively, the unfolding or conformational alteration of the core domain due to these mutations might provoke some aggregation of the mutants *in vivo*, thereby decreasing the efficiency of their recognition and degradation by MDM2.

p53 is subjected to an array of post-translational modifications that regulate its stability and consequently half-life that in turn influences the expression of p53 target genes. In particular phosphorylation generally results in its stabilization [4], [48], [49]. It has been reported that the phosphorylation status on T155 is important for the E6-mediated degradation of p53 in HeLa cells [37]. We observed that the replacement of T155 by aspartate protected p53 from HPV16 E6-mediated degradation but not when T155 was substituted by alanine or valine, and that all mutant proteins were still degraded by MDM2. When transfected in H1299 cells, p53-T155A and p53-T155V transactivated the p21 promoter but not p53-T155D. These data suggested a possible effect of the p53-T155D mutation on p53 fold. Indeed, while p53-T155A and, to some extent p53-T155V, were recognised by the Pab 1620 as the wild-type core domain, p53-T155D mutant was recognised by Pab 240 specific of “mutant” conformation. However, our observations regarding p53-T155V differ to previous work that indicated this mutant was stabilized in HeLa cells [37]. In our experiments p53-T155V was partially recognised by both antibodies Pab 1620 and 240. Moreover, according to 1D NMR spectra, this mutant adopted a folded conformation (data not shown). In addition, p53-T155V and HPV16 E6 coding vectors were co-transfected in H1299 p53-null cells, while in the previous study only p53-T155V mutant protein was ectopically expressed in HeLa cells. The increase in stability of p53-T155V observed by Bech-Otschir *et al.* may be due to a fraction of p53 produced with an altered conformation that was resistant to HPV18 E6 or to a titration of endogenous HPV18 E6.

Interestingly, the three unfolded mutants p53-Y107G, p53-T155D, and p53-L265A were still degraded by MDM2. Several studies positioned a second binding site for MDM2 in the core domain of p53 [35], [36], [41], [50], [51] but none of them used a mutagenesis approach. Our results suggested that changes induced by mutants p53-L265A, p53-Y107G, and p53-T155D in the folding of the core domain did not disturb the putative secondary docking site of MDM2 on p53 core domain nor inhibit MDM2-dependent ubiquitination of p53. In light of our observations we can hypothesize that the second binding site of MDM2 might be a flexible segment of the core domain, which does not require a fully folded context for binding to its partners.

While all core domains mutants of p53 remained susceptible to MDM2-mediated degradation regardless of their conformational status, we found that all the mutants which became protected against degradation by the viral E6 oncoprotein presented an unfolded or conformationaly altered core domain. On the one hand, this indicates that the conformation of the core domain is an essential parameter for the degradation of p53 by E6. On the other hand, it is possible that some of the residues which we have mutated participate in the binding interface between E6 and p53. However, at present we cannot draw any definite conclusion on this point. It is important to notice that, would we have skipped all the experiments aimed at analysing p53 folding and conformational status, we would have probably concluded that all the residues whose mutation inactivated the susceptibility of p53 to E6 degradation, were directly involved in the E6-p53 interface. This emphasizes the strategic importance of “fold-checking” biophysical experiments for the correct analysis of mutagenesis data, and also points to the limitations inherent to site-directed mutagenesis approach. To get definite answers on the E6-p53 interaction interface, we may have to wait for high-resolution structural studies of the E6-E6AP-p53 complex.

## Materials and Methods

### Plasmids

Using standard PCR protocols and the plasmid vector pCMV-neo-bam coding for p53 as the DNA template (pC53-SN3) [52], DNA sequences encoding the various forms of human p53 were obtained. These sequences were inserted in the expression vector plasmid pCDNA3-Amp. Several mutants were produced and named p53-T155A, p53-T155D, p53-T155V, p53-L264A, p53-L265A, p53-Y103G, p53-Y107G, p53-Y103G/Y107G, and p53-99-107 (where the region ^99^ SQKTYQGSY ^107^ was replaced by ^99^ GAGAGAGAG ^107^). HPV16 E6 sequence was inserted into pXJ40 allowing expression of E6 (158 amino acids) controlled by SV40 promoter. The expression vector pCDNA-E6K2 encoding the HPV16 E6 protein has previously been described by Giovane *et al.* [53]. For the transfection of MDM2, we used the expression vector pCOC-MDM2 described previously by Haupt *et al.* [54] and kindly provided by Professor Moshe Ohren (Weismann Institute, Rehovot, Israel).

GST fusion protein expression vectors were constructed using the pETM30 expression vectors kindly provided by Gunter Stier (EMBL, Heidelberg, Germany). DNA oligomers encoding the p53 wild-type and mutant core domain were inserted in to the pETM30, a modified pET24d vector containing a N-terminal His6-GST tag and a TEV protease cleavage site. This gave rise to the vectors pETM30-WTcore, pETM30-L265Acore, and pETM30-Y103Gcore.

### Cells and transfection

H1299 cells (p53-null human non-small-cell lung adenocarcinoma cells) were kindly provided by Professor Ingrid Hoffmann (DKFZ, Heidelberg, Germany) and were maintained in Dulbecco's modified Eagle's medium (DMEM) supplemented with 10% foetal bovine serum, Hepes and antibiotics. Various combinations of plasmids were transfected into the H1299 cells by use of lipofectamine (Invitrogen) according to the manufacturer's instructions. For the co-transfection assays, we used 1 µg of each plasmid, except for MDM2 where 5 times more was used.

### Western blot analysis

The level of p53, HPV16 E6, actin or MDM2 was determined by Western blotting. The cells were extracted in 150 mM NaCl, 50 mM tris HCl (pH 8), 5 mM EDTA, 1% NP40, 2 mM DTT and an antiprotease cocktail (Complete EDTA-free, Roche). After a short sonication step, the extract was clarified by centrifugation at 16000× g at 4°C for 20 min. Extracted proteins were then separated by 12% SDS-PAGE and analysed by Western blot. The membrane was incubated with different antibodies: mouse monoclonal anti-p53 antibody (DO-1, Calbiochem), rabbit polyclonal anti-p53 antibody (FL-393, Santa Cruz Biotechnology), rabbit polyclonal anti-p21 antibody (C-19, Santa Cruz Biotechnology), rabbit polyclonal anti-actin antibody (A2066, Sigma-Aldrich), mouse monoclonal anti-HPV16-E6 antibody (6F4, Euromedex) and analysed by enhanced chemiluminescence (Amersham Biosciences). For proteasome inhibitor assays, the cells were treated for 11 h with 100 µM of calpain inhibitor ALLN in 0.1% DMSO (Sigma-Aldrich). The cells were analysed 24 h after transfection.

### Immunoprecipitation analysis

Immunoprecipitation of p53 with antibodies Pab 1620 and Pab 240 (Calbiochem) was performed on H1299 total cell extracts. 25 µL of protein A sepharose resin was mixed on a wheel with one antibody for two hours at 4°C in buffer B (0.2% v/v NP40, 150 mM NaCl, 10 mM tris, pH 7.5 and 2 mM EDTA), and then the lysates containing the p53 mutant were added over night. Subsequently, beads were washed three times with buffer B, three times with buffer C (0.2% v/v NP40, 500 mM NaCl, 10 mM tris, pH 7.5, and 2 mM EDTA) and once with 10 mM tris, pH 7.5.

### *In vitro* degradation assay

^35^S-Labeled proteins were obtained by *in vitro*-translation using the TnT Quick Coupled Transcription/Translation rabbit reticulocyte lysate system (Promega) following the manufactures instructions and using Promix ^35^S-labeled Cysteine and Methionine (Promega). The lysates containing translated E6 protein (25 µL) and the indicated translated p53 protein (10 µL) were incubated at 28°C in the presence of 3 mM DTT, 100 mM NaCl, 25 mM Tris-HCl, pH 7.5 in a final volume of 50 µL. At the indicated times, 5 µL aliquots of these reactions were added to SDS sample buffer. Samples were separated by 12% SDS-PAGE and the gel was incubated for 45 min in fix buffer (20% v/v ethanol, 10% v/v acetic acid) and dried before exposure to a BioMax MR film (Kodak). Levels of radiolabeled p53 were quantified by densitometry (BIO-RAD, Quantity One Software).

### Luciferase reporter gene assays

H1299 cells were transfected with a p21-luciferase plasmid and with pCDNA-p53-WT or mutants. Twenty-four hours after transfection, the cells were washed with PBS and scrapped in 1 mL of ice-cold PBS. After centrifugation, the cell pellet was resuspended in 100 µL of buffer (100 mM KH_2_PO_4_, pH 7.8, 1 mM DTT) and incubated 20 min on ice and subsequently cleared by centrifugation at 16000× g. A Bradford assay was performed to determine the protein concentration of the cell lysates. 50 µL of the cell lysate were incubated with 50 µL of luciferase reaction buffer (100 mM KH_2_PO_4_, 6 mM ATP, 0.75 mM CoenzymeA, 15 mM Mg_2_S0_4_, 1 mM luciferine). The luciferase activity was then measured by a luminometer.

### Expression and purification of GST-p53 core domains

The core domain (residues 94–312 [45]) of the human p53 WT protein (p53core-WT) and the mutants p53-L265A (p53core-L265A) and p53-Y103G (p53core-Y103G) were cloned into pET30 and expressed in *E. coli* strain BL21 (DE3). Overexpression cultures were grown in unlabeled medium (CD) or in M9 minimal medium with ^15^N labeled NH_4_(SO_4_)_2_ as a sole nitrogen source (used for NMR ^1^H-^15^N correlation spectroscopy) at 37°C until OD_600_ reached 0.8. The cultures were then transferred to 20°C and induced with 0.5 mM isopropyl α-D-thiogalactoside (IPTG) over night. After induction of protein expression, bacteria were collected by centrifugation and resuspended in buffer (50 mM TrisHCl, 200 mM NaCl, 2 mM DTT, pH 6.8 supplemented with 2.5 µg/mL RNaseA, 2.5 µg/mL DNaseI and antiprotease Cocktail-Complete EDTA-free, Roche). The suspension was sonicated and centrifuged at 30000× g for 60 min. Supernatants were purified on a GST-Trap column (GE Healthcare) and the purified GST-p53 core was cleaved with recombinant TEV protease. p53 core domains were purified on a HiTrap-heparin column (GE healthcare) and by size exclusion on superdex 75 pg column (GE healthcare).

### NMR Spectroscopy

Samples of p53-WTc (100 µM), p53-L265Ac (70 µM) and p53-Y103Gc (125 µM) for NMR experiments were prepared in 20 mM phosphate buffer (pH 6.8), 200 mM NaCl, 2 mM DTT and 10% D_2_O. Approximately 250 µl of samples were transferred to 8 mm Shigemi tubes for data acquisition. In order to investigate the folding state of the p53 core domain, NMR experiments were conducted at 10°C on a Bruker DRX600 spectrometer equipped with a z-gradient triple resonance cryoprobe. ^1^H-^15^N SOFAST-HMQC [55] data were acquired with 1024 complex points and a spectral width of 4000 Hz in F_2_ (^1^H) and 128 complex points with 2400 Hz in F_1_ (^15^N). The recycle delay was 0.25 s and the number of transients used was 1024. Data were processed using XWINNMR (Bruker). Assignments are available within the BMRB ID 1TSR.

### Circular dichroism

All the CD experiments were followed with a Jasco J-815 spectropolarimeter (Easton, MD) fitted with an automatic 6-position Peltier thermostated cell holder. The instrument was calibrated with 10-camphorsulphonic acid. Far-UV CD data of p53core-Y103G and p53core-WT were obtained using a 0.1 mm path length cell (Quartz-Suprasil, Hellma UK Ltd) at 10.0°C±0.1°C. Spectra were acquired using a continuous scan rate of 50 nm/min and averaging at least ten scans. The response time was 2 sec. The absorbance of the sample and buffer were kept as low as possible: p53core-WT spectrum was carried out in 20 mM sodium phosphate (pH 6.8), 10 mM NaCl, 2 mM DTT and recorded between 180 to 260 nm. Due to the presence of precipitations of the p53core-L265A in low salt solution, the spectrum was recorded between 190 to 260 nm in 20 mM sodium phosphate (pH 6.8), 50 mM NaCl, 2 mM DTT. Each spectrum was at least repeated with fresh samples. All spectra were corrected by subtraction of the corresponding solvent spectrum obtained under identical conditions. The signal is expressed in mean residue ellipticity (deg.cm^2^.dmol^−1^). Thermal denaturation experiments were performed by following raw ellipticity at 210 nm (far UV) using a 1 mm path length cell (Quartz-Suprasil, Hellma UK, Ltd.). The temperature ranged from 10 to 80°C with steps of 0.4°C. The CD signal was integrated for 8 sec. Buffer conditions were 20 mM sodium phosphate (pH 6.8), 50 mM NaCl, 2 mM DTT. Thermal denaturation is irreversible due to protein precipitation.

## References

[1] Kastan MB, Zhan Q, el-Deiry WS, Carrier F, Jacks T (1992). A mammalian cell cycle checkpoint pathway utilizing p53 and GADD45 is defective in ataxia-telangiectasia.. Cell.

[2] Diller L, Kassel J, Nelson CE, Gryka MA, Litwak (1990). p53 functions as a cell cycle control protein in osteosarcomas.. Mol Cell Biol.

[3] May P, May E (1999). Twenty years of p53 research: structural and functional aspects of the p53 protein.. Oncogene.

[4] Bode AM, Dong Z (2004). Post-translational modification of p53 in tumorigenesis.. Nat Rev Cancer.

[5] Cho Y, Gorina S, Jeffrey PD, Pavletich NP (1994). Crystal structure of a p53 tumor suppressor-DNA complex: understanding tumorigenic mutations.. Science.

[6] Kastan MB, Onyekwere O, Sidransky D, Vogelstein B, Craig RW (1991). Participation of p53 protein in the cellular response to DNA damage.. Cancer Res.

[7] Maki CG, Huibregtse JM, Howley PM (1996). In vivo ubiquitination and proteasome-mediated degradation of p53(1).. Cancer Res.

[8] Brooks CL, Gu W (2004). Dynamics in the p53-Mdm2 ubiquitination pathway.. Cell Cycle.

[9] Yu ZK, Geyer RK, Maki CG (2000). MDM2-dependent ubiquitination of nuclear and cytoplasmic p53.. Oncogene.

[10] Haupt Y, Maya R, Kazaz A, Oren M (1997). Mdm2 promotes the rapid degradation of p53.. Nature.

[11] Whibley C, Pharoah PD, Hollstein M (2009). p53 polymorphisms: cancer implications.. Nat Rev Cancer.

[12] Evans SC, Viswanathan M, Grier JD, Narayana M, El-Naggar AK (2001). An alternatively spliced HDM2 product increases p53 activity by inhibiting HDM2.. Oncogene.

[13] Leach FS, Tokino T, Meltzer P, Burrell M, Oliner JD (1993). p53 Mutation and MDM2 amplification in human soft tissue sarcomas.. Cancer Res.

[14] Momand J, Zambetti GP, Olson DC, George D, Levine AJ (1992). The mdm-2 oncogene product forms a complex with the p53 protein and inhibits p53-mediated transactivation.. Cell.

[15] Soria C, Estermann FE, Espantman KC, O'Shea CC (2010). Heterochromatin silencing of p53 target genes by a small viral protein.. Nature.

[16] Dobner T, Horikoshi N, Rubenwolf S, Shenk T (1996). Blockage by adenovirus E4orf6 of transcriptional activation by the p53 tumor suppressor.. Science.

[17] Grand RJ, Parkhill J, Szestak T, Rookes SM, Roberts S (1999). Definition of a major p53 binding site on Ad2E1B58K protein and a possible nuclear localization signal on the Ad12E1B54K protein.. Oncogene.

[18] Huibregtse JM, Scheffner M, Howley PM (1993). Cloning and expression of the cDNA for E6-AP, a protein that mediates the interaction of the human papillomavirus E6 oncoprotein with p53.. Mol Cell Biol.

[19] Scheffner M, Werness BA, Huibregtse JM, Levine AJ, Howley PM (1990). The E6 oncoprotein encoded by human papillomavirus types 16 and 18 promotes the degradation of p53.. Cell.

[20] Werness BA, Levine AJ, Howley PM (1990). Association of human papillomavirus types 16 and 18 E6 proteins with p53.. Science.

[21] Nomine Y, Masson M, Charbonnier S, Zanier K, Ristriani T (2006). Structural and functional analysis of E6 oncoprotein: insights in the molecular pathways of human papillomavirus-mediated pathogenesis.. Mol Cell.

[22] Barbosa MS, Schlegel R (1989). The E6 and E7 genes of HPV-18 are sufficient for inducing two-stage in vitro transformation of human keratinocytes.. Oncogene.

[23] Grossman SR, Mora R, Laimins LA (1989). Intracellular localization and DNA-binding properties of human papillomavirus type 18 E6 protein expressed with a baculovirus vector.. J Virol.

[24] Schwarz SE, Rosa JL, Scheffner M (1998). Characterization of human hect domain family members and their interaction with UbcH5 and UbcH7.. J Biol Chem.

[25] Talis AL, Huibregtse JM, Howley PM (1998). The role of E6AP in the regulation of p53 protein levels in human papillomavirus (HPV)-positive and HPV-negative cells.. J Biol Chem.

[26] Scheffner M, Huibregtse JM, Vierstra RD, Howley PM (1993). The HPV-16 E6 and E6-AP complex functions as a ubiquitin-protein ligase in the ubiquitination of p53.. Cell.

[27] Liu Y, Kim BO, Kao C, Jung C, Dalton JT (2004). Tip110, the human immunodeficiency virus type 1 (HIV-1) Tat-interacting protein of 110 kDa as a negative regulator of androgen receptor (AR) transcriptional activation.. J Biol Chem.

[28] Be X, Hong Y, Wei J, Androphy EJ, Chen JJ (2001). Solution structure determination and mutational analysis of the papillomavirus E6 interacting peptide of E6AP.. Biochemistry.

[29] Gu J, Rubin RM, Yuan ZM (2001). A sequence element of p53 that determines its susceptibility to viral oncoprotein-targeted degradation.. Oncogene.

[30] Li X, Coffino P (1996). Identification of a region of p53 that confers lability.. J Biol Chem.

[31] Li X, Coffino P (1996). High-risk human papillomavirus E6 protein has two distinct binding sites within p53, of which only one determines degradation.. J Virol.

[32] Lechner MS, Laimins LA (1994). Inhibition of p53 DNA binding by human papillomavirus E6 proteins.. J Virol.

[33] Thomas M, Massimi P, Jenkins J, Banks L (1995). HPV-18 E6 mediated inhibition of p53 DNA binding activity is independent of E6 induced degradation.. Oncogene.

[34] Mansur CP, Marcus B, Dalal S, Androphy EJ (1995). The domain of p53 required for binding HPV 16 E6 is separable from the degradation domain.. Oncogene.

[35] Shimizu H, Burch LR, Smith AJ, Dornan D, Wallace M (2002). The conformationally flexible S9–S10 linker region in the core domain of p53 contains a novel MDM2 binding site whose mutation increases ubiquitination of p53 in vivo.. J Biol Chem.

[36] Yu GW, Rudiger S, Veprintsev D, Freund S, Fernandez-Fernandez MR (2006). The central region of HDM2 provides a second binding site for p53.. Proc Natl Acad Sci U S A.

[37] Bech-Otschir D, Kraft R, Huang X, Henklein P, Kapelari B (2001). COP9 signalosome-specific phosphorylation targets p53 to degradation by the ubiquitin system.. Embo J.

[38] Stewart D, Kazemi S, Li S, Massimi P, Banks L, Koromilas AE (2004). Ubiquitination and proteasome degradation of the E6 proteins of human papillomavirus types 11 and 18.. J Gen Virol.

[39] Kussie PH, Gorina S, Marechal V, Elenbaas B, Moreau J (1996). Structure of the MDM2 oncoprotein bound to the p53 tumor suppressor transactivation domain.. Science.

[40] Picksley SM, Vojtesek B, Sparks A, Lane DP (1994). Immunochemical analysis of the interaction of p53 with MDM2;–fine mapping of the MDM2 binding site on p53 using synthetic peptides.. Oncogene.

[41] Gu J, Chen D, Rosenblum J, Rubin RM, Yuan ZM (2000). Identification of a sequence element from p53 that signals for Mdm2-targeted degradation.. Mol Cell Biol.

[42] Pavletich NP, Chambers KA, Pabo CO (1993). The DNA-binding domain of p53 contains the four conserved regions and the major mutation hot spots.. Genes Dev.

[43] Wang PL, Sait F, Winter G (2001). The ‘wildtype’ conformation of p53: epitope mapping using hybrid proteins.. Oncogene.

[44] Stephen CW, Lane DP (1992). Mutant conformation of p53. Precise epitope mapping using a filamentous phage epitope library.. J Mol Biol.

[45] Wong KB, DeDecker BS, Freund SM, Proctor MR, Bycroft M (1999). Hot-spot mutants of p53 core domain evince characteristic local structural changes.. Proc Natl Acad Sci U S A.

[46] Rudiger S, Freund SM, Veprintsev DB, Fersht AR (2002). CRINEPT-TROSY NMR reveals p53 core domain bound in an unfolded form to the chaperone Hsp90.. Proc Natl Acad Sci U S A.

[47] Bullock AN, Fersht AR (2001). Rescuing the function of mutant p53.. Nat Rev Cancer.

[48] Olsson A, Manzl C, Strasser A, Villunger A (2007). How important are post-translational modifications in p53 for selectivity in target-gene transcription and tumour suppression?. Cell Death Differ.

[49] Saito S, Yamaguchi H, Higashimoto Y, Chao C, Xu Y (2003). Phosphorylation site interdependence of human p53 post-translational modifications in response to stress.. J Biol Chem.

[50] Burch LR, Midgley CA, Currie RA, Lane DP, Hupp TR (2000). Mdm2 binding to a conformationally sensitive domain on p53 can be modulated by RNA.. FEBS Lett.

[51] Wallace M, Worrall E, Pettersson S, Hupp TR, Ball KL (2006). Dual-site regulation of MDM2 E3-ubiquitin ligase activity.. Mol Cell.

[52] Baker S J, Markowitz S, Fearon ER, Willson JK, Vogelstein B (1990). Suppression of humain colorectal carcinoma cell growth by wild-type p53.. Science.

[53] Giovane C, Trave G, Briones A, Lutz Y, Wasylyk B (1999). Targetting of the N-terminal domain of the humain papillomavirus type 16 E6 oncoprotein with monomeric ScFvs blocks the E6-mediated degradation of cellular p53.. J Mol Recognit.

[54] Haupt Y, Barak Y, Oren M (1996). Cell type-specific inhibition of p53-mediated apoptosis by mdm2.. Embo J.

[55] Schanda P, Kupce E, Brutscher B (2005). SOFAST-HMQC experiments for recording two-dimensional heteronuclear correlation spectra of proteins within a few seconds.. J Biomol NMR.

